# Persistent post-traumatic headache vs. migraine: an MRI study demonstrating differences in brain structure

**DOI:** 10.1186/s10194-017-0796-0

**Published:** 2017-08-22

**Authors:** Todd J. Schwedt, Catherine D. Chong, Jacob Peplinski, Katherine Ross, Visar Berisha

**Affiliations:** 1 Mayo Clinic Arizona, 5777 East Mayo Boulevard, Phoenix, AZ 85255 USA; 2 Phoenix VA Health Care System, Phoenix, USA; 3 Arizona State University, Phoenix, USA

**Keywords:** Post-traumatic headache, Migraine, Traumatic brain injury, Magnetic resonance imaging, Brain structure, Brain volume, Brain curvature, Brain surface area, Cortical thickness

## Abstract

**Background:**

The majority of individuals with post-traumatic headache have symptoms that are indistinguishable from migraine. The overlap in symptoms amongst these individuals raises the question as to whether post-traumatic headache has a unique pathophysiology or if head trauma triggers migraine. The objective of this study was to compare brain structure in individuals with persistent post-traumatic headache (i.e. headache lasting at least 3 months following a traumatic brain injury) attributed to mild traumatic brain injury to that of individuals with migraine.

**Methods:**

Twenty-eight individuals with persistent post-traumatic headache attributed to mild traumatic brain injury and 28 individuals with migraine underwent brain magnetic resonance imaging on a 3 T scanner. Regional volumes, cortical thickness, surface area and curvature measurements were calculated from T1-weighted sequences and compared between subject groups using ANCOVA. MRI data from 28 healthy control subjects were used to interpret the differences in brain structure between migraine and persistent post-traumatic headache.

**Results:**

Differences in regional volumes, cortical thickness, surface area and brain curvature were identified when comparing the group of individuals with persistent post-traumatic headache to the group with migraine. Structure was different between groups for regions within the right lateral orbitofrontal lobe, left caudal middle frontal lobe, left superior frontal lobe, left precuneus and right supramarginal gyrus (*p* < .05). Considering these regions only, there were differences between individuals with persistent post-traumatic headache and healthy controls within the right lateral orbitofrontal lobe, right supramarginal gyrus, and left superior frontal lobe and no differences when comparing the migraine cohort to healthy controls.

**Conclusions:**

In conclusion, persistent post-traumatic headache and migraine are associated with differences in brain structure, perhaps suggesting differences in their underlying pathophysiology. Additional studies are needed to further delineate similarities and differences in brain structure and function that are associated with post-traumatic headache and migraine and to determine their specificity for each of the headache types.

## Background

The majority of individuals with post-traumatic headache (PTH) have headache characteristics that are consistent with a migraine phenotype [1–4]. The only clinical feature that then differentiates PTH from migraine is the head injury itself. The International Classification of Headache Disorders (ICHD) 3 beta criteria stipulate that a PTH must begin within 7 days of the head injury or within 7 days of being able to detect headaches following a head injury [5]. If the PTH persists for at least 3 months, it is classified as “persistent PTH” (PPTH). The ICHD criteria do not include any headache characteristics that differentiate PTH from other headache types such as migraine or tension-type headache. For the patient without a history of migraine who has head trauma and develops headache immediately following the trauma, the diagnosis of PTH is rather straightforward. However, even in such a situation, it is not clear if the head trauma caused a unique headache type (i.e. PTH) with a unique underlying pathophysiology or if the trauma unmasked an underlying propensity toward the development of migraine. Identification of differences in the pathophysiology of migraine and PTH would support the notion that migraine and PTH are truly distinct headache types.

The objective of this study was to compare measures of brain regional volume, cortical thickness, surface area, and brain curvature in a cohort of patients with PPTH attributed to mild traumatic brain injury (mTBI) to a cohort of patients with migraine. Differences in brain structure would serve as evidence that PPTH and migraine might be associated with different underlying mechanisms and thus should indeed be considered distinct headache types.

## Methods

### Research participants

Men and women between the ages of 18 and 65 were enrolled. A certified headache specialist assigned headache diagnoses according to the criteria in the ICHD 3 beta [5]. Individuals with PPTH attributed to mTBI were excluded if they had a personal history of moderate or severe TBI, migraine or any other headache type prior to their head injury (infrequent episodic tension-type headache was allowed). Individuals with migraine were excluded if they had a personal history of TBI. Individuals with PPTH or migraine were eligible for this study even if they used headache treatments (abortive, preventive) and even if they had comorbid conditions such as depression, anxiety, and post-traumatic stress disorder. Healthy control subjects had no history of migraine or other headache type (other than infrequent tension-type headache) and no history of TBI. Participants were enrolled from patient clinics at Mayo Clinic and at the Phoenix VA Health Care System. Healthy controls were enrolled from the Phoenix area via advertisements and word-of-mouth referrals.

### Questionnaires

All research participants provided information regarding their demographics and were screened for TBI using the Ohio State TBI Identification method [6]. Individuals with mTBI provided information about the injury mechanism. Participants with migraine and those with PPTH provided detailed information about their headaches. All participants completed the Beck Depression Inventory and the State-Trait Anxiety Inventory [7–9].

### Magnetic resonance imaging

All participants were imaged on a 3-Tesla MAGNETOM Skyra MRI scanner located at the Mayo Clinic Arizona Hospital. Structural sequences included a high-resolution 3D T1-weighted sagittal magnetization prepared rapid gradient echo (MP-RAGE) series (TE (echo time) =3.03 ms; TR (repetition time) = 2.4 s; 1x1x1.3 mm voxels; 256 × 256 mm field-of-view (FOV), acquisition matrix 256 × 256) and T2-weighted images in axial plane (TE = 84 ms; TR = 6800 ms; 1x1x4 mm voxels; 256x256mm FOV, acquisition matrix 256 × 256). T1 data were used for calculating brain regional volumes, surface area, cortical thickness and curvature. T2 images were used in conjunction with T1 images to rule-out gross anatomical abnormalities. Three participants were excluded due to gross anatomical abnormalities (one PPTH participant with white matter hyperintensities, one migraine participant with generalized cerebral atrophy, and one healthy control with focal parietal atrophy).

T1 MP-RAGE data processing was performed using FreeSurfer (version 5.3, http://surfer.nmr.mgh.harvard.edu/). All image post-processing was performed on one Mac computer running OS X Lion 10.7.5 software [10]. FreeSurfer methodology, which is discussed more fully in prior publications, includes skull stripping, automated Talairach transformation, segmentation of subcortical gray and white matter, intensity normalization, and gray-white mater boundary tessellation and surface deformation [11–14]. MRI data were used to determine regional cortical and subcortical volumes, cortical surface areas, cortical thicknesses and brain curvature over the left and right hemispheres. For quality control, the automated segmentations and parcellations of each individual participant were manually inspected for errors before including the data for statistical analysis. Mean thickness, surface area, volume, and curvature estimates were extracted from FreeSurfer and exported to MATLAB (2007a, MathWorks) for further analyses. In total, there were 86 regions for which measures of volume were obtained and 68 regions for which measures of cortical thickness, surface area, and curvature were obtained. To account for differences in overall brain size amongst individuals, volume measurements were normalized using total intracranial volume, and area measurements were normalized using total surface area.

### Statistical analyses

Descriptive statistics were used to describe demographic data, scores on questionnaires, and headache characteristics. Two-tailed t-tests or Fisher’s exact tests were utilized for comparing these data between subject cohorts. An independent samples t-test was performed on each MRI brain metric (i.e. regional volumes, cortical thicknesses, area, curvature) to identify significant differences between PPTH and migraine cohorts. To control for covariates (nuisance variables) that could contribute to the variances between PPTH and migraine, an analysis of covariance (ANCOVA) was performed on each brain MRI metric that significantly differed between patient groups via t-test. For the PPTH vs. migraine analysis, ANCOVA controlled for sex, age, depression scores, state and trait anxiety scores, and the number of years a subject had been having headaches, since these were parameters for which there were differences between the PPTH and migraine groups. For brain regions in which a structural measure differed between PPTH and migraine cohorts after controlling for nuisance variables in the initial ANCOVA: 1) correlations between the measurement with the number of years with PTH and with the number of total lifetime TBIs were calculated; 2) ANCOVA was performed to determine if there were differences in brain structure of these regions between PPTH and healthy controls; and 3) ANCOVA was performed to determine if there were differences in brain structure of these regions between migraine and healthy controls. In the ANCOVA that compared PPTH with healthy controls, we controlled for sex, depression scores, and anxiety scores, since the PPTH and healthy control cohorts differed on these parameters. In the ANCOVA that compared migraine with healthy controls, we controlled for depression and anxiety scores, since the migraine and healthy control cohorts differed on these variables. For all analyses, the statistical significance threshold was set to *p* < 0.05.

## Results

Eighty-four subjects, consisting of 28 subjects with PPTH, 28 with migraine, and 28 healthy controls were included (Table 1). Average age of subjects was 35.9 +/− 9 years and 56% were female. Comparing the patients with PPTH to those with migraine, there were no differences in age (PPTH: 35.1 +/− 9.6 years vs. Migraine: 37.5 +/− 8.5 years, *p* = .33) or headache frequency (16.6 +/− 7.8 days per month vs. 16.4 +/− 8.1 days per month, *p* = .93). There were differences in sex (PPTH: females 32% vs. Migraine: 68%, *p* = .02), number of years with headache (8.5 +/− 7.8 years vs. 17.1 +/− 9.3 years, p = <.001), depression scores (18.6 +/− 9 vs. 6.1 +/− 5.3, p = <.001), state anxiety scores (39.3 +/− 14.4 vs. 32 +/− 7.9, *p* = .02) and trait anxiety scores (47.2 +/− 12.6 vs. 36.4 +/− 9.8, p = <.001). The healthy control group had an average age of 35.2 +/− 9.1 years, 68% were female, average depression score was 1.5 +/− 1.9, average state anxiety score was 24.9 +/− 6, and average trait anxiety score was 27.4 +/− 5.5. Compared to the PPTH cohort, the healthy control cohort had similar age (*p* = .97), fewer females (*p* = .02), and lower depression (*p* < .001), state anxiety (*p* < .001), and trait anxiety scores (*p* < .001). Compared to the migraine cohort, the healthy control cohort had similar age (*p* = .33), the same number of females, and lower depression (*p* < .001), state anxiety (*p* < .001), and trait anxiety scores (*p* < .001).

**Table 1:** Participant characteristics

	PPTH (*n* = 28)	Migraine (*n* = 28)	PPTH vs. migraine *p*-value	Healthy controls (*n* = 28)	PPTH vs. healthy controls *p*-value	Migraine vs. healthy controls *p*-value
Age in years (mean +/− SD)	35.1 +/− 9.6	37.5 +/− 8.5	.33	35.2 +/−9.1	.97	.33
Sex (female:male)	9:19	19:9	.02	19:9	.02	1
Headache Days/Month (mean +/− SD)	16.6 +/− 7.8	16.4 +/− 8.1	.93	N/A	N/A	N/A
Years with Headache (mean +/− SD)	8.5 +/− 7.8	17.1 +/− 9.3	<.001	N/A	N/A	N/A
State Anxiety (mean +/− SD)	39.3 +/− 14.4	32 +/− 7.9	.02	24.9 +/− 6	<.001	<.001
Trait Anxiety (mean +/− SD)	47.2 +/− 12.6	36.4 +/− 9.8	<.001	27.4 +/− 5.5	<.001	<.001
Beck Depression Inventory (mean +/− SD)	18.6 +/− 9	6.1 +/− 5.3	<.001	1.5 +/− 1.9	<.001	<.001

*PPTH* persistent post-traumatic headache, *SD* standard deviation, *N/A* not applicable

Amongst the 28 individuals with PPTH, 21 (75%) had a phenotype that was consistent with a diagnosis of migraine (had the symptoms not been initiated by TBI), 4 with probable migraine, 2 with tension-type headache, and 1 was unclassifiable. Twenty-seven reported that their headaches were of moderate or severe intensity, 25 reported sensitivity to sound, 24 sensitivity to light, 22 nausea, 21 throbbing quality of headache, 19 worsening of headache with physical activity, and 11 vomiting. Five subjects had one TBI in their lifetime, 13 had 2 TBIs, two had 3 TBIs, one had 4 TBIs, two had 5 TBIs, and five had 6 or more TBIs. Of the TBIs leading to PPTH, 14 were due to explosions/blasts, 7 were due to sports injuries, 4 were due to motor vehicle accidents, and 3 were due to falls.

**Fig. 1:**
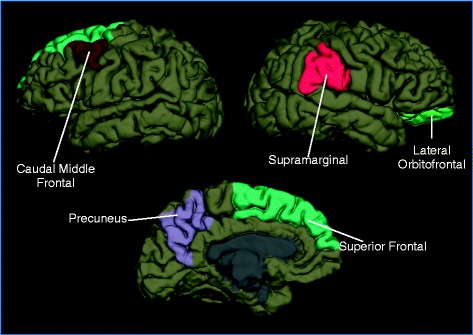
Regions with structural differences when comparing individuals with persistent post-traumatic headache (PPTH) to those with migraine. When comparing structural measurements of entire brain regions in patients with PPTH to patients with migraine, the right lateral orbital frontal region differed in area, volume, and curvature. The left caudal middle frontal, precuneus, and superior frontal regions and the right supramarginal gyrus region differed in cortical thickness

Comparison of the PPTH cohort to the migraine cohort revealed differences in area of the right lateral orbitofrontal area (*p* = .008), curvature of the right lateral orbital frontal area (*p* = .019), volume of the right lateral orbital frontal area (*p* = .025), and thickness of the left caudal middle frontal region (*p* = .025), left precuneus (*p* = .041), right supramarginal gyrus (*p* = .041), and left superior frontal region (*p* = .048). (Table 2, Fig. 1) For each of these regions, thickness, area and/or volume measurements in those with PPTH were less than those within the migraine cohort. Lateral orbital frontal region curvature was greater in the PPTH cohort compared to the migraine cohort, suggesting underlying structural damage. The significance of each variable within the ANCOVA is reported in Table 3. Amongst those regions that differed in structure when comparing PPTH to migraine, there were no significant correlations between the numbers of lifetime TBIs or years with PTH with brain structure.

**Table 2:** Brain regions with structural differences when comparing individuals with persistent post-traumatic headache (PPTH) to those with migraine

Region	PPTH mean (SD)	Migraine mean (SD)	PPTH vs. migraine *p*-value	Healthy control mean (SD)	PPTH vs. healthy controls *p*-value	Migraine vs. healthy controls *p*-value
Right Lateral Orbitofrontal Area	.0287 (.0029)	.0301 (.0005)	*.008*	.0315 (.0018)	*.024*	.62
Right Lateral Orbitofrontal Curvature	.1610 (.0092)	.1545 (.0163)	*.019*	.1590 (.0085)	.075	.54
Right Lateral Orbitofrontal Volume	.0045 (.0005)	.0048 (.0006)	*.025*	.0052 (.0012)	.15	.75
Left Caudal Middle Frontal Thickness	2.3787 (.1648)	2.5027 (.1245)	*.025*	2.5535 (.3769)	.066	.78
Left Precuneus Thickness	2.2948 (.0898)	2.3746 (.0460)	*.041*	2.3845 (.1633)	.17	.30
Right Supramarginal Thickness	2.4909 (.0651)	2.5916 (.2086)	*.041*	2.5675 (.1110)	*.001*	.55
Left Superior Frontal Thickness	2.5637 (.0339)	2.6788 (.1909)	*.048*	2.7275 (.3188)	*.035*	.83

Volume and area measurements are normalized. Thickness measurements are in mm. Curvature measurements are in mm^−1^

Italicized *p*-values are significant (*p*<.05)

**Table 3:** Significance of each variable in the ANCOVA for brain region measurements that differed between PPTH and migraine

Region	Age	Depression	State anxiety	Trait anxiety	Years with PTH	Sex
Right Lateral Orbitofrontal Area	.898	*.043*	.674	.580	.448	.810
Right Lateral Orbitofrontal Curvature	.419	.847	.374	.117	.183	.746
Right Lateral Orbitofrontal Volume	.067	.130	.985	.340	.187	*.033*
Left Caudal Middle Frontal Thickness	*.029*	.521	.065	*.004*	.266	.140
Left Precuneus Thickness	.055	.440	.205	*.023*	.065	.874
Right Supramarginal Thickness	*.000*	.401	.371	.072	.612	.914
Left Superior Frontal Thickness	*.010*	.547	.586	.093	.567	.251

Italicized *p*-values are significant (*p*<.05)

To better interpret the differences in brain structure identified between PPTH and migraine, the seven regional structural measures that differed between the PPTH and migraine cohorts were compared between the PPTH group to a group of healthy controls and between the migraine group and the healthy controls. Amongst these regions, there were differences between the PPTH group and healthy controls for thickness of the right supramarginal gyrus (*p* = .001), area of the right lateral orbitofrontal region (*p* = .024), and thickness of the left superior frontal region (*p* = .035). For all of these comparisons, PPTH was associated with less area and cortical thickness compared to the healthy controls. Comparisons between the migraine group and the healthy control group demonstrated no significant differences in the seven structural measurements that differed when comparing the PPTH group to the migraine group.

## Discussion

The phenotypic similarities between migraine and PPTH justify the need for studies that investigate mechanistic similarities and differences between migraine and PPTH. The main finding of this study is that there are differences in brain structure between patients who have PPTH and those with migraine, perhaps suggesting that these two headache types are associated with distinct underlying pathophysiology despite their substantial similarities in symptoms.

Brain areas that differed between PPTH and migraine were located in lateral orbitofrontal, superior and middle frontal, precuneus and supramarginal gyrus regions. The observation that only three of the seven brain regional measurements differed when comparing PPTH to healthy controls and none of the seven brain regional measurements differed when comparing migraine to healthy controls suggests specificity of some brain structural differences to the comparison of PPTH vs. migraine. We are not aware of prior studies that have compared brain structure or function in a group of individuals with PPTH to a group with migraine and thus are unable to compare our findings to others. However, there are a few studies that have compared brain structure and metabolism in individuals with PTH compared to healthy controls. For example, a longitudinal voxel-based morphometry study by Obermann and colleagues compared 32 patients with PTH attributed to whiplash injury to healthy controls [15]. During the first 14 days post-TBI, there were no differences in brain structure. However, at 3 months, PTH was associated with decreased gray matter density in the anterior cingulate cortex and dorsolateral prefrontal cortex. As headaches subsided by 1 year, the gray matter density normalized in these brain areas. Amongst those who developed PPTH, at 1 year there was in increase in gray matter in the midbrain, thalamus and cerebellum. Assuming that the patients who developed PPTH and those who did not had similar brain injuries, this study supports the notion that brain structural changes in patients with PPTH are related to the continued headaches and are not due solely to the inciting brain injury. A magnetic resonance spectroscopy study of 17 individuals with PTH attributed to mTBI (9 with acute PTH and 8 with PPTH) found that compared to healthy controls those with PTH had metabolic abnormalities in the anterior frontal lobes, anterior and posterior medial frontal lobes, and medial parietal lobes [16]. Although there was little power in this study to detect differences between patients with acute PTH compared to those with PPTH, those with PPTH had non-significant but lower N-acetylaspartate values (a marker for neuronal health). In our study, there were no significant correlations between the structural measurements of regions that differed when comparing PPTH to migraine with the number of years that individuals had PPTH. However, our patient population differed than those in prior publications in that our patients had a longer duration of PPTH at the time of imaging (mean of 8.5 years). Studies of mTBI have also demonstrated abnormalities of brain structure in several of the regions that we identified to have different structure in PPTH compared to migraine [17–19]. Most of these previously published studies have not reported whether the participants had PTH and they did not attempt to disentangle the effects of the brain injury itself on brain structure vs. the effects of post-TBI symptoms on brain structure.

The brain regions that differed in structure when comparing individuals with PPTH to those with migraine in this study have all been previously demonstrated to participate in pain processing. Frontal regions play roles in the affective and cognitive evaluation of pain [20, 21]. Frontal regions have previously been found to have abnormal structure, function, and functional connectivity in individuals with different headache types including migraine, cluster headache, and medication overuse headache [22–26]. The precuneus, a core region of the default mode network that is responsible for self-referential processing and interoception, participates in the determination of pain sensitivity and pain thresholds [27–30]. Prior studies have implicated the precuneus and the default mode network in headache disorders including migraine, medication overuse headache, and cluster headache [22, 30–33]. The supramarginal gyrus is likely involved in cognitive evaluation of pain including pain empathy [34, 35]. The supramarginal gyrus has previously been demonstrated to have atypical function and structure in groups of individuals with migraine and medication overuse headache [36, 37]. Although there are sufficient data to support a role for these brain regions in pain processing and headache, the explanation as to why the structure of these regions differs in individuals with PPTH compared to those with migraine is yet to be elucidated. It is plausible that these are brain regions that are simply more susceptible to the effects of mTBI and once damaged they contribute to the initiation and persistence of PTH.

The different measures used in this study (volume, area, cortical thickness, curvature) provide complementary information about brain structure. Regional volumes, surface area, cortical thickness and curvature are plastic measures of brain structure that commonly show alterations in the presence of aging, learning, and neurodegeneration related to disease. Brain curvature provides a measure of cortical folding, with increased curvature indicating areas of sharper cortical folds. Increased cortical curvature has been associated with white matter damage, aging, neurodegenderative disease, and mild TBI [38]. Although regional volumes have been most commonly used to measure brain structure and compare brain structure between subject cohorts, measurements of area and cortical thickness are likely to be more sensitive to small changes in brain structure. Regional brain area, cortical thickness, and volume have been previously identified to differ in cohorts of individuals with headache compared to healthy controls and to contribute to subclassification of headache types [39–41].

Limitations: Similar to individuals with migraine and PPTH in the community and in clinical practice, our research participants had co-morbid medical conditions, and they were utilizing medications. For example, patients with PPTH had higher anxiety and depression scores than patients with migraine and healthy controls. Although we controlled for many potentially confounding variables in our ANCOVA, including depression and anxiety scores, it is not entirely possible to decouple the effects of these co-morbid illnesses on brain structure from the effects of migraine and PPTH on brain structure. However, study results would be of little value in understanding migraine and PPTH if we excluded patients with typical disease and only enrolled the very rare individual who has migraine or PPTH in isolation. Inclusion of typical patients with migraine and PPTH makes the results from this study generalizable to the typical individual with migraine or PPTH. Furthermore, the subject cohorts were not exactly matched for age and sex. Although we attempted to do so, the realities of our patient populations resulted in enrolling more men with PPTH (mostly armed forces veterans) and more women with chronic migraine. Sex was controlled for in our ANCOVA and was significant for only one of the brain measurements that differed between migraine and PPTH, but it still could have impacted our results to some extent. Perhaps the biggest limitation of this analysis is the inability to differentiate the effects of TBI from that of PPTH on brain structure. It is not clear if the differences in brain structure identified in this study are due to PPTH or if the differences would be similar in individuals who have persistent post-TBI symptoms without headache. We are currently trying to enroll those rare patients who do not have headache but do have other persistent symptoms following a mild TBI for comparison to the PPTH group.

## Conclusions

In conclusion, although there was substantial overlap in symptoms between individuals with PPTH and those with migraine, the structure of several brain regions differed in individuals with PPTH compared to those with migraine. These structural differences suggest that the pathophysiology of PPTH might be different than that of migraine and support the classification of PPTH and migraine as distinct headache types. Additional studies are planned to confirm these imaging findings and to determine their specificity.

## Abbreviations

ANCOVA: Analysis of covariance
FOV: Field of view
ICHD: International classification of headache disorders
MP-RAGE: Magnetization prepared rapid gradient echo
mTBI: Mild traumatic brain injury
N/A: Not applicable
PPTH: Persistent post-traumatic headache
PTH: Post-traumatic headache
SD: Standard deviation
TBI: Traumatic brain injury
TE: Echo time
TR: Repetition time
